# Inverting Monotonic Nonlinearities by Entropy Maximization

**DOI:** 10.1371/journal.pone.0165288

**Published:** 2016-10-25

**Authors:** Jordi Solé-Casals, Karmele López-de-Ipiña Pena, Cesar F. Caiafa

**Affiliations:** 1 Data and Signal Processing Research Group, U Science Tech, University of Vic – Central University of Catalonia, Vic, Catalonia, Spain; 2 Systems Engineering and Automation Department, Universidad del País Vasco/Euskal Herriko Unibertsitatea, EleKin Research Group, Donostia, Spain; 3 Instituto Argentino de Radioastronomía (IAR), CONICET, CCT – La Plata, Buenos Aires, Argentina; Technische Universitat Darmstadt, GERMANY

## Abstract

This paper proposes a new method for blind inversion of a monotonic nonlinear map applied to a sum of random variables. Such kinds of mixtures of random variables are found in source separation and Wiener system inversion problems, for example. The importance of our proposed method is based on the fact that it permits to decouple the estimation of the nonlinear part (nonlinear compensation) from the estimation of the linear one (source separation matrix or deconvolution filter), which can be solved by applying any convenient linear algorithm. Our new nonlinear compensation algorithm, the MaxEnt algorithm, generalizes the idea of Gaussianization of the observation by maximizing its entropy instead. We developed two versions of our algorithm based either in a polynomial or a neural network parameterization of the nonlinear function. We provide a sufficient condition on the nonlinear function and the probability distribution that gives a guarantee for the MaxEnt method to succeed compensating the distortion. Through an extensive set of simulations, MaxEnt is compared with existing algorithms for blind approximation of nonlinear maps. Experiments show that MaxEnt is able to successfully compensate monotonic distortions outperforming other methods in terms of the obtained Signal to Noise Ratio in many important cases, for example when the number of variables in a mixture is small. Besides its ability for compensating nonlinearities, MaxEnt is very robust, i.e. showing small variability in the results.

## 1. Introduction

Nonlinear models are powerful tools for modelling practical situations when linear models fail. This is the case of post-nonlinear (PNL) source separation problems and nonlinear blind deconvolution scenarios. In real world situations, usually we do not have access to the distortion input. Hence we cannot use traditional methods, which assume that both the input and the output of the distortion are available [1]. Examples of such traditional methods are those based on higher-order input/output cross-correlation [2], bispectrum estimation [3, 4] or on the application of the Bussgang and Prices theorems [5, 6] for nonlinear systems with Gaussian inputs. In this work, we will focus only on blind identification methods.

**Fig 1:**
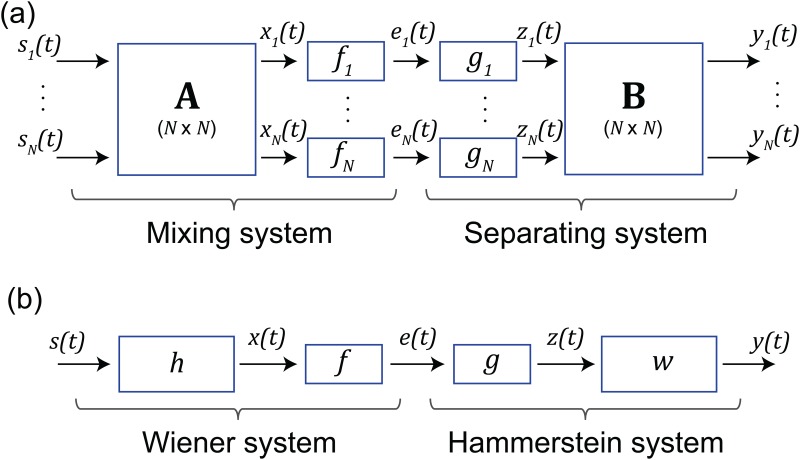
**Typical applications where nonlinear compensation is required:** (a) the mixing—separating system for the source separation problem with PNL mixtures and (b) the Wiener-Hammerstein system for deconvolution problem with PNL distortion.

Blind source separation with PNL mixtures (see Fig 1a), requires estimating the inverses of the nonlinear maps *f*_*n*_ and the inverse of the mixing matrix **A**(linear part). This can be done by minimizing the Mutual Information (MI) of the inversion structure output, i.e. by solving an optimization problem over the parameters of the linear and nonlinear parts together, as detailed in [7]. However, this leads to complex and slow algorithms.

On the other hand, in a single channel scenario, there is a particular class of nonlinear systems composed by a linear filter followed by a memoryless nonlinear distortion (Fig 1b). This class of nonlinear systems, also known as a Wiener system, is not only a nice and mathematically attracting model, but also a realistic model used in various areas, such as biology [8], industry [9], sociology and psychology (see also [10] and the references therein). A fully blind procedure for finding the inverse system (Hammerstein system), when the (unknown) input signal *s*(*t*) is a non-Gaussian i.i.d. process, is detailed in [11, 12]. If the input process is not i.i.d. but is a linear filtered version of an i.i.d. noise (the so-called innovation), the output *y*(*t*) provides the innovation instead of the input signal. The restitution of the input then requires the prior knowledge (or the estimation) of the filter generating the signal *s*(*t*), as detailed in [12].

A simple and very fast method for roughly estimating the inverse of a nonlinear map has been proposed in [11] and used in [12], which will be referenced here as the *Gaussianization* algorithm (see **Section 2.1**). This method is very attractive because it focuses only on the nonlinear compensation estimation. After the nonlinear compensation is found, we can solve the linear part (inverse matrix or deconvolution filter) by applying any available linear algorithm. This nonlinear-linear decoupling approach allows one to obtain simpler and faster algorithms compared to global algorithms that estimate the linear and nonlinear parts together [7,11,12].

The basic idea for the Gaussianization algorithm is to notice that signals *x*_*n*_(*t*) in Fig 1a and *x*(*t*) in Fig 1b, i.e. just before the nonlinear map, are a weighted sum of random variables, which in the case of variables with finite second order moments, is more Gaussian than the individual inputs. In fact, according to the Central Limit Theorem, the random variable *X*_*n*_ tends toward a Gaussian variable as *N* → ∞ (for finite second order moments). Note that we use capital letters to refer to a random variable associated to a signal. For example, a signal *x*(*t*) is considered as a time series (realizations) obtained from a random variable *X*. The nonlinear mapping function *f*_*n*_ changes the distribution, and consequently we can assume that the random variable *E*_*n*_ = *f*_*n*_(*X*_*n*_) is more distant from a Gaussian than *X*_*n*_. Then, the algorithm estimates the inverse of *f*_*n*_ as the nonlinear mapping function *g*_*n*_ which enforces the random variable *Z*_*n*_ = *g*_*n*_(*E*_*n*_) to be exactly Gaussian.

In this paper we will focus only on the nonlinear compensation, assuming a monotonic (unknown) distortion, generalizing the idea of Gaussianization for estimating the inverse of the nonlinear map by using maximization of entropy strategy instead. We provide a sufficient condition under which the output of a nonlinear transform has smaller entropy compared with its input, thus giving a guarantee for the MaxEnt method to succeed inverting the nonlinear distortion. The new algorithm consists in adjusting a nonlinear mapping function *g*_*n*_, through an appropriate parameterization, so that the entropy of *Z*_*n*_ = *g*_*n*_(*E*_*n*_) is maximum under the constraint of unit variance. Although the idea is similar to that used in [13], it differs since the recovered random variable *z* (after nonlinear inversion) has not necessarily a Gaussian distribution, but maximum entropy. In **Section 2** we explain the theoretical principles of the Gaussianization and Uniformization algorithms and introduce the equations for our new proposed method: the MaxEnt algorithm. In **Section 3**, we present extensive experimental results showing the robustness and performance of our new method compared to the Gaussianization and Uniformization methods and we compare MaxEnt against a state-of-the-art global optimization based method. In **Section 4**, our main conclusions are outlined.

## 2. Materials and Methods

In this article we denote random variables by capital letters and their realizations by the corresponding lower case letter. For example, *X* and *x*, correspond to a random variable and its realization (value), respectively.

Under the assumption that a random variable *X* is close to a Gaussian, if we let *E* = *f*(*X*), then we may estimate the inverse *g* = *f*^−1^ by enforcing *Z* = *g*(*E*) to be Gaussian [13]. We can generalize this idea by observing that entropy at the output of the linear system *X* is always higher than the entropy of each independent input *S*_*n*_, under the constant variance constraint (see Fig 1a). Additionally, if we assume that the nonlinear distortion has the effect to decrease the entropy, then we can estimate the inverse *g* = *f*^−1^ searching, over the space of plausible nonlinear maps, the one that makes the entropy of *Z* = *g*(*E*) to be maximum under the unit-variance constraint. We call this new method as the MaxEnt nonlinear compensation method and it is described in detail in **Section 2.2**.

It is well known that the maximum entropy distribution for unbounded support under mean and variance constraints is the Gaussian distribution, whereas for bounded supports under no (but adding-up) constraint is the uniform distribution. However, it is important to note that, by using a Maximum Entropy algorithm (MaxEnt) we are not enforcing directly to obtain a Gaussian or uniform distribution for *Z*. However, our extensive experimental results (see Section 3) confirm the fact that for most important cases, MaxEnt outperforms Gaussianization and Uniformization strategies and gives also more robust results.

In the following sections, the theoretical foundations are presented and the algorithms are derived for the Gaussianization, Uniformization and MaxEnt methods.

### 2.1. Gaussianization and Uniformization

The simplest approach for computing the inverse system *g* by Gaussianization [13] is based on the property of the cumulative density function (cdf). Consider the random variable *E*, and denote its cdf *F*_*E*_(*u*) = P_r_(*E* < *u*) where P_r_ denotes the probability. The random variable *U* = *F*_*E*_(*E*) is then uniformly distributed in [0, 1]. Denoting by Φ(*u*) the Gaussian cdf, which transforms a unit variance Gaussian variable into a uniform random variable in [0, 1], it is clear that Φ^−1^(*U*)is a unit variance Gaussian random variable. Then, a simple approximation of the inverse *g* of the nonlinear mapping function *f* is $\hat{g}=\Phi^{-1}\circ F_{E}$ [13,14].

In the case of variables with bounded support and no constraints, the maximum entropy is achieved by the uniform distribution. Therefore, we also consider Uniformization as a possible way to compute the inverse *g*. The same strategy explained for Gaussianization, but using the cdf for the uniform distribution is then used for computing the monotonous nonlinear mapping *g*.

### 2.2. Entropy maximization (MaxEnt)

We assume that the nonlinear transformation *E* = *f*(*X*), has the effect to decrease the normalized (unit-norm) entropy of the input, i.e. *H*(*E*) < *H*(*X*), so the transform can be blindly estimated by MaxEnt. We know this is true when the input variable *X* is of maximum entropy, e.g. Gaussian, but it is not clear a priori, under which conditions on the distribution of *X* and the nonlinearity *f*(∙) the MaxEnt method is well posed. The following theorem states a sufficient condition for the decrease of entropy under a nonlinear transformation. As we demonstrate in **Section 3.7**, through experimental validation, this condition holds true in most practical cases.

***Theorem 1 (sufficient condition for entropy decrease under a nonlinear transform)***: *Given a zero-mean and unit-variance variable X and a monotonic and antisymmetric nonlinear function f*(∙), *if the following condition holds true*:

$${\text{E}}^{2}\left[f^{\prime }\left(\text{x}\right)\right]<\text{E}\left[f^{2}\left(\text{x}\right)\right]$$ (1)

*then the entropy of the normalized output variable E* = *f*(*X*) *is smaller than the input variable X*.

***Proof*: *see*** Appendix

In order to understand the consequences of this result, the following corollary, provides the theoretical justification of the MaxEnt method for a particular family of nonlinearities, found in many practical applications [15].

***Corollary 1*:*(particular case*** *f*(*x*) = *x*^3^ *+ βx with β* ≥ 0):

*For any probability density function (pdf) p*_*X*_(*x*) *and the parameterized nonlinear function f*(*x*) = *x*^3^ *+ βx*, *the entropy of the normalized output variable E* = *f*(*X*) *is smaller than the input variable X in the following cases*:

1. *Super-Gaussian or zero excess kurtosis* (*μ*_4_ ≥ 3), *μ*_6_ > 9 *and any β* ≥ 0;
2. *Super-Gaussian* (*μ*_4_ > 3), *μ*_6_ < 9 *and β* > 0.5(9 − *μ*_6_)/(*μ*_4_ −3);
3. *Sub-Gaussian* (*μ*_4_ < 3), *μ*_6_ > 9 *and β* < 0.5(*μ*_6_ − 9)/(3 −*μ*_4_);
  *where μ*_*p*_ *is the moment of order p*. *It is noted that*, *in our case with standardized variables* (E(*X*) = 0 *and* E[*X*^2^]), *μ*_4_ *and μ*_6_ *correspond to the kurtosis and the hyper-flatness statistical measures*, *respectively*.

***Proof*: *see*** Appendix

It is important to highlight that this corollary gives us a guarantee for the MaxEnt method to work for the case of for super-Gaussian or zero excess kurtosis variables when *μ*_6_ > 9 and give us clear conditions on the sixth order moment *μ*_6_ and parameter *β* for the case of sub-Gaussian variables.

#### 2.2.1. Derivation of the MaxEnt algorithm

Consider the entropy of the continuous unit variance random variable *Z* = *g*(*E*):

$$H\left(Z\right)=-\text{E}\left[\text{log}\left(p_{Z}\left(z\right)\right)\right]$$ (2)

where *p*_*Z*_(*z*) denotes the pdf of random variable *Z* = *g*(*E*). By using a well-known basic property of the entropy, *H*(*Z*) can be written as follows:

$$H\left(Z\right)=H\left(E\right)+\text{E}\left[\text{log}\left|g^{\prime }\left(e\right)\right|\right]$$ (3)

We can consider different ways to parameterize the nonlinearity *g*(*e*) and maximize eq (3) in terms of the used parameterization. In the following sections, two different parameterizations are proposed: polynomial and neural network parameterizations.

#### 2.2.2. Polynomial parameterization

One of the simplest parameterization options consists in using a polynomial. This will give us a very simple algorithm, with very few parameters.

Let us consider a *K* degree polynomial for *g*(*e*):

$$g\left(e\right)=\sum_{k=0}^{K}a_{k}e^{k}.$$ (4)

Then, its derivative with respect to *e* is:

$$g^{\prime }\left(e\right)=\sum_{k=0}^{K}a_{k}ke^{k-1}=a^{T}e\;,$$ (5)

where **a** = (*a*_1_, *a*_2_, …, *a*_*K*_)^*T*^ and **e** = (1,2*e*, …, *Ke*^*K*−1^)^*T*^. By using eq (5) into eq (3) we arrive at the following expression for the entropy:

$$H\left(Z\right)=H\left(E\right)+\text{E}\left[log\left(\left|a^{T}e\right|\right)\right]$$ (6)

And the gradient of this expression with respect to the vector of parameters **a** (polynomial coefficients) is:

$$\nabla_{a}H=\text{E}\left[\frac{e}{a^{T}e}\right],$$ (7)

where, by assuming that underlying random processes are wide-sense stationary and ergodic, the expectation can be computed by averaging over time, i.e. by a sample mean estimator.

Finally, we propose an iterative constrained gradient algorithm to estimate the inverse mapping g by repeating the following steps until a convergence criterion or maximum number of iterations is reached:

$$\begin{array}{ll} a\leftarrow a+\mu \nabla_{a}H & ;\text{steepest}\;\text{ascend}\;\text{step}\\ a\leftarrow a{/}_{\sigma_{Z}} & \;;\text{enforce}\;\text{unit}-\text{variance}, \end{array}$$ (8)

where *μ* is the stepsize parameter and *σ*_*Z*_ is the standard deviation of the compensated signal *Z* = *g*(*E*).

Polynomial parameterization is very simple, but may have problems when is used for inverting functions whose inverses are not well approximated by a low order polynomial. To avoid working with high order polynomials, in the following section, we propose a nonlinearity parameterization based on a neural network.

#### 2.2.3. Neural network parameterization

Another interesting possibility is to use neural networks, and specifically multi-layer perceptrons (MLP) [16]. As MLP is well known for approximating any continuous and bounded function, it is a good candidate for estimating the inverse function of *f*, if it exists.

The model of *g*(*e*) using a multilayer perceptron with one hidden layer of *K* units can be written as follows:

$$g\left(e\right)=\sum_{k=1}^{K}a_{k}\sigma \left(c_{k}e-b_{k}\right),$$ (9)

where *a*_*k*_, *b*_*k*_ and *c*_*k*_ are the weight of the output, the bias and the weight of the input parameters for each unit of the neural network, respectively, and *σ*(*t*) = (1 + *e*^−*t*^)^−1^ is a sigmoid function [16]. Then, its derivative with respect to *e* is:

$$g\prime \left(e\right)=\sum_{k=1}^{K}a_{k}c_{k}\sigma \prime \left(c_{k}e-b_{k}\right)={\left(a\circ c\right)}^{T}\theta ,$$ (10)

Where **a** = (*a*_1_, *a*_2_, …, *a*_*K*_)^*T*^, **b** = (*b*_1_, *b*_2_, …, *b*_*K*_)^*T*^, **c** = (*c*_1_, *c*_2_, …, *c*_*K*_)^*T*^, **θ** = (*θ*_1_, *θ*_2_, …, *θ*_*K*_)^*T*^ with, *θ*_*k*_ = *σ*′(*c*_*k*_*e* − *b*_*k*_) and ‘∘’ stands for the entry-wise (Hadamard) product of vectors. By using eq (10) in eq (3) we obtain the following expression of the entropy:

$$H\left(Z\right)=H\left(E\right)+\text{E}\left[log\left(\left|{\left(a\circ c\right)}^{T}\theta \right|\right)\right],$$ (11)

whose gradients with respect to **a**, **b** and **c** are:

$$\begin{array}{c} \nabla_{a}H=\text{ E}\left[\frac{c\circ \theta }{{\left(a\circ c\right)}^{T}\theta }\right],\\ \nabla_{b}H=\text{E}\left[\frac{a\circ c\circ \varphi }{{\left(a\circ c\right)}^{T}\theta }\right],\\ \nabla_{c}H=\text{ E}\left[\frac{a\circ \theta +a\circ c\circ \varphi \;e}{{\left(a\circ c\right)}^{T}\theta }\right], \end{array}$$ (12)

where ***φ*** = (*φ*_1_, *φ*_2_, …, *φ*_*K*_)^*T*^ with *φ*_*k*_ = *σ*′′(*c*_*k*_*e* − *b*_*k*_). Finally, we propose to use a constrained gradient ascend algorithm as follows:

$$\begin{array}{ll} a\leftarrow a+\mu_{a}\nabla_{a}H & ;\text{ steepest ascend step for vector }a\\ b\leftarrow b+\mu_{b}\nabla_{b}H & ;\text{ steepest ascend step for vector }b\\ c\leftarrow c+\mu_{c}\nabla_{c}H & ;\text{ steepest ascend step for vector }c\\ a\leftarrow a{/}_{\sigma_{Z}} & ;\text{ enforce unit-variance}, \end{array}$$ (13)

where *μ*_**a**_, *μ*_**b**_ and *μ*_**c**_ are the stepsize parameters and *σ*_*Z*_ is the standard deviation of the compensated signal *Z* = *g*(*E*).

## 3. Experimental Results and Discussion

In order to evaluate the results obtained with the new proposed method, in this section we compute the performances obtained with the Gaussianization method [13], the Uniformization method (similar to [13] but enforcing uniform distribution for *Z*) and MaxEnt (the new proposed algorithm). The inversion performance for all the methods is calculated as the Signal to Noise Ratio (SNR), which is defined as follows:

$$SNR=10\text{ log}\left(\frac{\text{E}\left[z^{2}\right]}{\text{E}\left[{\left(z-x\right)}^{2}\right]}\right)\;.$$ (14)

It is noted that our gradient search optimization method with both types of parameterizations, i.e. polynomial and neural network, may suffer from stacking at local minima. To alleviate the local minima problem, the initialization for vector **a** (polynomial case) and vectors **a**,**b**,**c** (neural network case) are chosen such that the initial guess of the inversion function is the identity, i.e. $\hat{g}\left(x\right)=x$. For example, the initialization in the polynomial parameterization case is as follows: *a*_1_ = 1, and *a*_*k*_ = 0 ∀ *k* ≠ 1.

Taking into account that we can deal with many different scenarios, in the following sections we explore how different conditions and/or values of the parameters can affect the results of the nonlinear compensation. We analyze the effect of the number of samples (*T*), the mixing matrix (**A**), the number of sources (*N*) and the nonlinearity type. For the sake of simplicity, MaxEnt only with polynomial parameterization along with Gaussianization and Uniformization are used in **Sections 3.1–3.4**. In all these experiments, the maximum number of iterations for the MaxEnt algorithm is *N*_*iter_max*_ = 100. In **Section 3.5**, we present a detailed analysis of the performance obtained by the MaxEnt algorithm with polynomial and neural network parameterizations. In **Section 3.6**, we compare MaxEnt against a *state-of-the-art* methods and, in **Section 3.7**, we experimentally evaluate the sufficient condition of Theorem 1 for different source distributions and nonlinearities.

### 3.1. Effect of the number of samples (*T*)

**Fig 2:**
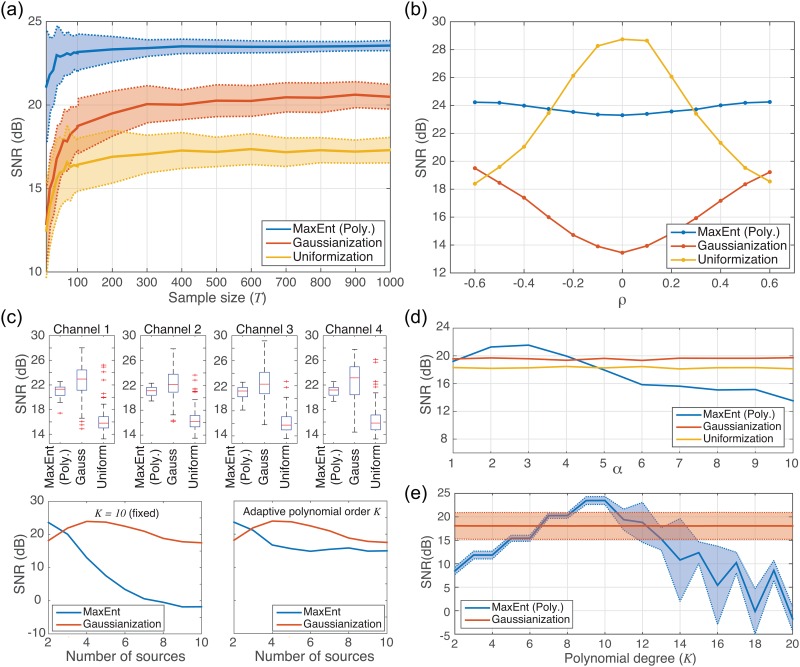
**Experimental results with MaxEnt algorithm with polynomial (Poly.) and neural network (N.N.) parameterizations, Gaussianization and Uniformization methods, for different scenarios over a set of 100 experiments:** (a) Mean ± std SNR evolution (channel 1) when the number of samples *T* changes from 10 to 1000 for a fixed random matrix **A**. (b) Mean SNR evolution (channel 1) when the 2 × 2 mixing matrix **A** changes according to parameter *ρ* as in eq (14). (c) Top: Boxplot of SNR for each channel with a fixed 4 × 4 random mixing matrix. Bottom: SNR as a function of the number of mixing sources for a fixed polynomial order *K* = 10 (left) and for adaptively chosen polynomial order *K*. (d) Mean SNR versus nonlinearity parameter α as in eq (15). (e) Mean ± std SNR of MaxEnt (Poly.) as a function of the polynomial degree (*K*) compared against the SNRs obtained with the Gaussianization algorithm.

In order to evaluate the effect of the number of available samples *T*, we make experiments by fixing the order of the polynomial to *K* = 10 and by using a random matrix **Aϵℝ**^**2**×**2**^. Sources were generated using zero-mean and unit-variance continuous uniformly distributed random variables, which determines the $\left[-\sqrt{3},+\sqrt{3}\right]$ support. Nonlinearities *f*_1_(*x*) = *f*_2_(*x*) = *tanh*(3*x*) + 0.1*x* are used. When we use a mixing matrix **A** the support is increased, therefore there is a strong effect of the nonlinearities. The number of samples ranged from *T* = 10 to *T* = 1,000. The experiments were repeated 100 times and the mean and standard deviation of SNRs were computed. In terms of mean values, as we can see in Fig 2a, the MaxEnt algorithm outperforms the Gaussianization (approx. +3dB) and the Uniformization (approx. +6dB) methods. Moreover, MaxEnt algorithm reaches the maximum SNR value (approx. 23dB) quicker than the other two algorithms. It is noted that the variance decreases with large *T* for all methods. Interestingly, for a very small number of samples *T* < 100, Gaussianization has lower variance than MaxEnt.

In the following experiments we fix the number of samples to *T* = 500.

### 3.2 Effect of the mixing matrix (A)

Another important parameter is the (unknown) mixing matrix **A**. In order to analyze how the mixing matrix affects the performance of the nonlinear compensation, we parameterize a general mixing matrix **A**, for the case of two sources/observations, as follows:

$$A=\left[\begin{array}{cc} 1 & \rho \\ \rho & 1 \end{array}\right]$$ (15)

We did experiments by tuning the parameter *ρ* from −0.6 < *ρ* < +0.6, for a fixed number of samples *T* = 500, using uniformly distributed continuous random variables as source signals, setting the order of the polynomial to *K* = 10 and fixing the nonlinearities as *f*_1_(*x*) = *f*_2_(*x*) = *tanh*(3*x*) + 0.1*x*. The obtained averaged SNRs in channel 1, over 100 repeated experiments, are shown in Fig 2b. It is noted that for *ρ* = 0 there is no mixing process, thus the observed signals are directly the distorted (through *f*(*x*)) original sources. We observe that MaxEnt is stable along the changes on the matrix **A**. On the other hand, it is also interesting to notice the performance of the Gaussianization and Uniformization algorithms: when there is (almost) no mixture (|*ρ*| close or equal to 0), Gaussianization does not work but Uniformization does because sources have an exact uniform distribution. However, it is important to note that in practice sources are not uniform, hence for practical situations Uniformization would not give such perfect solutions.

### 3.3 Results with *N* ≥ 4 sources

Until now and for the sake of simplicity, we have presented results of the inversion of nonlinearities for the case of mixtures with only two sources. If we have more mixing sources, we tend to a better situation according to the Central Limit Theorem, therefore we should expect good results for Gaussianization algorithm. First, we performed experiments mixing *N* = 4 random uniformly distributed continuous sources and using 100 different (random) mixing matrices **A** (with ones in the main diagonal). We fixed the order of the polynomial to *K* = 10 and nonlinearities as *f*(*x*) = *tanh*(3*x*) + 0.1*x*. Fig 2c **(top)** presents the obtained SNRs (dB) for all 100 experiments in each channel as a box plot. We can see that Gaussianization algorithm obtains best-averaged results (about 23 dB in channel 1) but with a very high variance (about 2.5 dB), while MaxEnt provides an average performance a little bit lower (< 1 dB less), with a very small variance (about 1 dB). So, the MaxEnt algorithm is the best compromise. It is highlighted that, by using the Gaussianization and Uniformization methods, the results have a considerably larger variance compared to the MaxEnt algorithm. In other words, MaxEnt is more robust because the algorithm maximizes entropy independently of the distribution of the mixture. The Uniformization method gives the worst results because mixing more sources makes the mixture to be farther away from the uniform distribution.

Finally, we analyzed the performance of MaxEnt and Gaussianization as a function of the number of mixing sources, with the nonlinearity *f*(*x*) = *tanh*(3*x*) + 0.1*x*. and using 100 different (random) mixing matrices **A** (with ones in the main diagonal). Fig 2c **(bottom-left)** shows the obtained SNRs (dB) for the case of using a fixed degree of the polynomial to *K* = 10, whereas in Fig 2c **(bottom-right)** the adaptive strategy for setting the polynomial degree *K* was used (see section 3.4). It is noted that there is a trade-off between Gaussianization and MaxEnt. For more input sources, Gaussianization is better since polynomial limit the accuracy of Maxent and, for less and non-Gaussian input sources, Gaussianization is less precise.

### 3.4 Effect of the nonlinearities

The type of nonlinearity plays also an important role. Using polynomials, it is expected that our method will be successful only when the inverse of the monotonic nonlinear function is well approximated by a finite degree polynomial. Here, we explore how the polynomial parameterization deals with a family of functions of the form

f(x)=tanh(α·x)+0.1x ,    α =1, 2, …, 10. (16)

**Fig 3:**
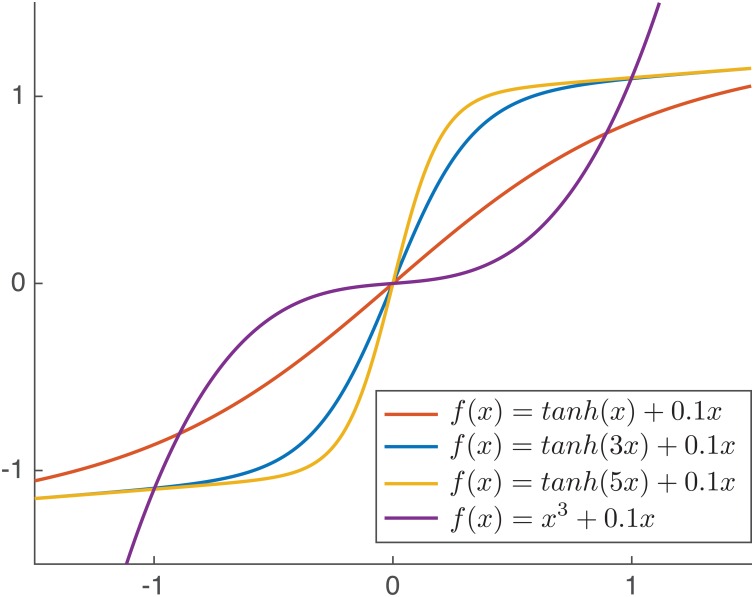
Family of nonlinearities used in the experiments.

For α = 1 the nonlinear function is almost linear, while for α ≥ 5 the nonlinear function is highly saturating the input signal (see Fig 3). Therefore, the inverse will be a polynomial of low degree in the first case and a polynomial of higher degree in the second case. To deal with this optimal selection of the polynomial, we have computed the objective function E[*log*(|**a**^*T*^**e**|)] (eq (6)) for a wide range, e.g. degree 2 to degree 15, and selected the best degree value *K* for the polynomial i.e. the one that provides largest entropy. In Fig 2c **(bottom**), we compare the results of using a fixed polynomial degree (*K* = 10, left) against using the dynamic selection of polynomial degree (right) as a function of the number of mixing sources.

Fig 2d shows the results for MaxEnt, Gaussianization and Uniformization for all types of nonlinearities of eq (16). It is noted that MaxEnt outperforms the rest of algorithms for 2 ≤ *α* ≤ 4. When *α* = 1 the performance of MaxEnt (mean SNR = 19.21dB, std = 0.26) is comparable with Gaussianization (mean SNR = 19.56, std = 0.97). However, when *α* > 4 it the performance of MaxEnt drops because the polynomial parameterization is not able to adapt to the high-saturating shape of the distortion. It is noted also that the degree of the polynomial selected by the algorithm changes according to the complexity of the function to be inverted: for smooth functions (*α* = 1) the selected degree is small (*K* = 4), while for a harder distortion (*α* = 5) the selected degree is higher (*K* = 12). According to these results, in the following sections, we fix the parameter *α* = 3 and the degree of the polynomial to *K* = 10.

### 3.5. Effect of sources distributions and nonlinearities on the MaxEnt performance with polynomial and neural network parameterization

In this set of experiments we explore the performance obtained by MaxEnt algorithm with polynomial and neural network parameterizations for two different types of nonlinearities: *f*(*x*) = *tanh*(3*x*) + 0.1*x* and *f*(*x*) = *x*^3^ + 0.1*x* (see Fig 2). More specifically, we compare our approach against the Gaussianization and Uniformization methods using two types of sources: uniformly distributed continuous random noise and (3-level) discrete random noise (–0.4, 0, +0.4). Based on the analysis of the previous set of experiments, we fix the parameters according to Table 1.

**Table 1:** Parameter selection for experiments with polynomial parameterization (1 case) and with neural network parameterization (2 cases).

	Polynomial	Neural network (I)	Neural network (II)
**Mixing matrix**	$A=\left[\begin{array}{cc} 1 & 0.6\\ 0.7 & 1 \end{array}\right]$	$A=\left[\begin{array}{cc} 1 & 0.6\\ 0.7 & 1 \end{array}\right]$	$A=\left[\begin{array}{cc} 1 & 0.6\\ 0.7 & 1 \end{array}\right]$
**Nonlinear functions**	*f*(*x*) = *tanh*(3*x*) + 0.1*x*	*f*(*x*) = *tanh*(3*x*) + 0.1*x*	*f*(*x*) = *x*^3^ + 0.1*x*
**Sample size**	*T* = 500	*T* = 500	*T* = 500
**Model order**	*K* = 10	*K* = 7	*K* = 7
**Maximum number of iterations**	*N*_*iter_max*_ = 100	*N*_*iter_max*_ = 50,000	*N*_*iter_max*_ = 50,000
**Adaptation step**	*μ* = 0.1	*μ* = 10	*μ* = 0.001
**Sources type**	Discrete or continuous (uniformly distributed)	Discrete or continuous (uniformly distributed)	Discrete or continuous (uniformly distributed)
**Number of experiments**	*N*_*exp*_ = 100	*N*_*exp*_ = 20	*N*_*exp*_ = 20

**Fig 4:**
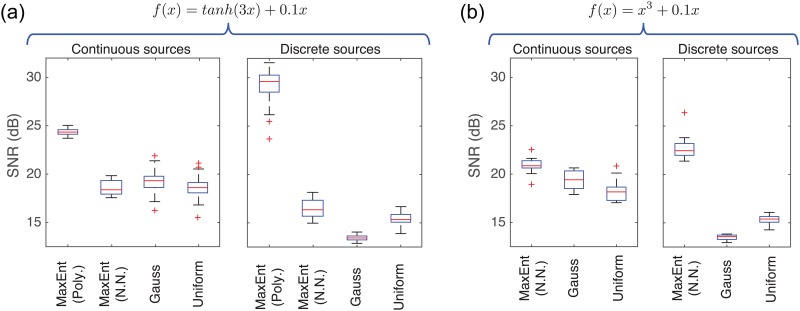
SNR (dB) boxplots obtained in channel 1 for MaxEnt algorithm with polynomial (Poly.) and neural network (N.N.) parameterizations, Gaussianization and Uniformization methods, for continuous (uniformly distributed) and (3-level) discrete sources (–0.4, 0, +0.4). (a) Results for the nonlinearity *f*(*x*) = *tanh*(3*x*) + 0.1*x*. (b) Results for the nonlinearity *f*(*x*) = *x*^3^ + 0.1*x* (polynomial parameterization is not included here because MaxEnt was not able to well approximate the nonlinearity showing convergence problems).

We have repeated the experiments 100 times for the polynomial parameterization and 20 times for the neural network parameterization. Fig 4 shows statistics of SNR improvement in dB obtained with all the methods in all the situations. In this figure we observe that MaxEnt with polynomial parameterization gives always the best SNR (dB) (Fig 4a) having smaller variance in the case of continuous sources compared to the rest of the methods. On the other hand, neural network parameterization gives similar results as Gaussianization and Uniformization for the case of continuous sources but it outperforms those methods for the case of discrete sources (Fig 4a and 4b), especially for when *f*(*x*) = *x*^3^ + 0.1*x*. It is noted that discrete sources do not work as well as continuous sources under the Gaussianization because a summation of two 3-state random variables results in a 7- to 9-state random variable, and this will hardly approximate a Gaussian random variable when only two variables are summed. It is a mere consequence of the asymptotic character of the central limit theorem that is not met in this setting; hence maximal entropy methods should succeed better.

It is important to note that the polynomial parameterization cannot approximate well the inverse of the nonlinear function *f*(*x*) = *x*^3^ + 0.1*x*, so the MaxEnt algorithm didn’t converge resulting in erroneous solutions. For this reason, MaxEnt (Poly) was not included in Fig 4b. On the other hand, neural network parameterization works very well for this nonlinearity outperforming the Uniformization and Gaussianization methods.

**Fig 5:**
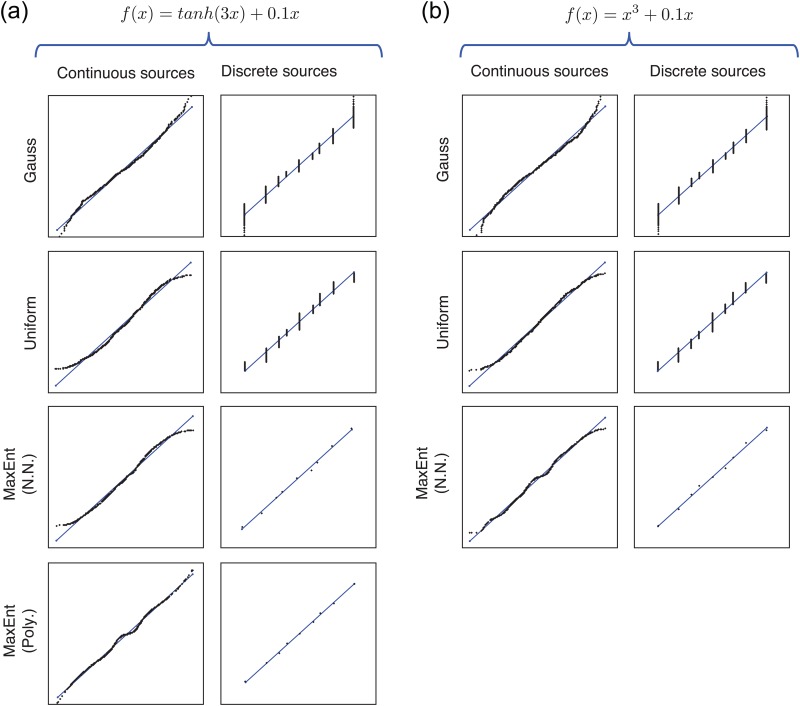
Examples of obtained nonlinear compensation for each of the tested algorithms for continuous (uniformly distributed) and (3-level) discrete sources (–0.4, 0, +0.4). (a) Results for the nonlinearity *f*(*x*) = *tanh*(3*x*) + 0.1*x*. (b) Results for the nonlinearity *f*(*x*) = *x*^3^ + 0.1*x*.

Examples of the nonlinear compensations obtained by all the methods in these experiments are shown in Fig 5 in the form of *x*(*t*) versus *z*(*t*) scatter-plot. In order to visually compare the performance, the identity function is superimposed to each curve. It is interesting to note that Gaussianization, Uniformization and MaxEnt with neural networks provide less accurate compensations at the edges, while MaxEnt with polynomial parameterization does in the central region.

### 3.6 Comparison of MaxEnt against a state-of-the-art global optimization based algorithm

In order to demonstrate the validity of our algorithm, we present here some simulation results comparing our method combined with the classical JADE algorithm [17], the MaxEnt+JADE algorithm (JADE Matlab code available in the author’s webpage: http://perso.telecom-paristech.fr/~cardoso/Algo/Jade/jadeR.m), against Mutual Information Minimization (MIM) algorithm proposed in [7] (MIM Matlab code provided by Prof. Christian Jutten, GIPSA Lab.), which implements a global optimization, i.e. by inverting at the same time the nonlinear and linear parts. We used the same parameters as in section 3.5 (see Table 1, Polynomial case), for uniformly distributed continuous sources.

Numerical SNR (dB) results as (mean±std) are shown in Table 2. We can observe that both strategies are very similar in terms of the obtained global mean SNR, but our new method is more robust (smaller variance). Also, it is interesting to note that our algorithm is much better than MIM regarding the inversion of non-linear part, so we suspect that the global performance could be further improved by using other algorithm for the linear part, for which there are a bunch of alternatives in the literature.

**Table 2:** SNR (dB) as mean±std obtained in 100 experiments. Nonlinear part and global system inversion results are presented, for MaxEnt+JADE and MIM algorithms.

	Non-linear part inversion	Non-linear part inversion	Global system inversion	Global system inversion
	New method	MIM method	New method	MIM method
**Channel 1**	24.30±0.32	21.48±1.57	17.22±0.39	16.85±1.99
**Channel 2**	24.00±0.37	20.74±1.37	16.29±0.61	15.91±2.40

Regarding the associated computational cost, we obtained an improvement of one order of magnitude. More specifically, in this example, our method requires 0.28 seconds in average for inverting the global system, while MIM method spent 2.86 seconds in average. MIM computational time increases exponentially with *T* while our algorithm has a linear dependency on *T*, so the difference will be higher with longer signals.

**Fig 6:**
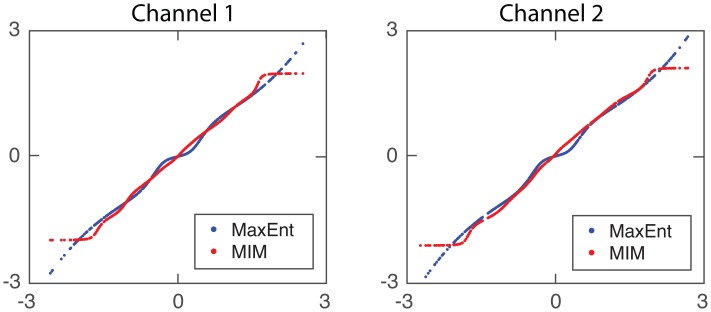
Nonlinear compensation of nonlinearities for MaxEnt algorithm (blue line) and MIM algorithm (red line). Channel 1 is depicted in the left part, channel 2 in the right part.

In Fig 6 we present an example of nonlinear compensation using our MaxEnt method (blue line) and MIM (red line), for channel 1 (left) and channel 2 (right).

Finally, it is important to note also that our nonlinear inversion strategy allow us to use any linear BSS algorithm. We have only tried with JADE but any other algorithm can be used, and this could potentially increase the final performance.

### 3.7 Experimental evaluation of the sufficient condition in Theorem 1

In Table 3, we evaluated experimentally the condition of eq (1) by averaging *f*^2^(*x*) and *f*′(*x*) over *T* = 10,000 samples, using several probability distributions *p*_*X*_(*x*) and various nonlinearities. As expected, the condition holds true for the case of a Gaussian distribution (second column). Also, as expected the condition does not hold true for the case of a variable *X* being the sum of two uniformly distributed variables (third column) and *f*(*x*) = *x*^3^ + *βx*, because in this case *μ*_4_ < 3 and *μ*_9_ < 9 (see Corollary 1. Case II). However, our experimental results in section 3.5, shows that even when the condition does not hold true in this case, the MaxEnt method successfully invert the nonlinear transform.

**Table 3:** Experimental evaluation of the sufficient condition in Theorem 1. T = 10,000 samples where used and experiments where repeated N = 500 times. The significance p value (H0: E^2^[f′(X) > E[f^2^(X)]) in all the cases where p≪0.0001. The fourth and sixth order moments were computed also experimentally. Cases for which the sufficient condition does not hold true are displayed in bold type (< 0).

	Gauss	*x*_1_ + *x*_2_, *x*_*p*_ unif.	$x_{1`}^{2}+x_{2`}^{2}$, *x*_*p*_ unif.	$x_{1`}^{3}+x_{2`}^{3}$, *x*_*p*_ unif.
***μ***_**4**_	3.0	2.4	2.5	2.8
***μ***_**6**_	14.9	7.5	10.4	15.3
***f***(***x***)	**E**[***f***^**2**^(**X**)]−**E**^**2**^[***f***′(**X**)]			
*tanh*(*x*) + 0.1*x*	> 0	> 0	> 0	> 0
*tanh*(3*x*) + 0.1*x*	> 0	> 0	> 0	> 0
*tanh*(5*x*) + 0.1*x*	> 0	> 0	> 0	> 0
*x*^3^ + 0.1*x*	> 0	**< 0**	> 0	> 0
*x*^3^ + 0.3*x*	> 0	**< 0**	> 0	> 0
*x*^3^ + 0.5*x*	> 0	**< 0**	> 0	> 0

Besides, we generated also samples for a variable *X* being the sum of squared and cubic uniformly distributed variables in order to have a wider range of fourth and sixth order moments (fourth and fifth columns). In all these cases the sufficient condition held true, which gives us a guarantee that the MaxEnt works in these cases.

## Conclusions

In this paper, we proposed a generalization of the Gaussianization/Uniformization method for blind estimation of a nonlinear map. The method is based on the entropy maximization of the nonlinear outputs in order to approximate the unknown nonlinear function. We provide a sufficient condition on the probability distribution and the nonlinear distortion that gives a guarantee for the MaxEnt method to succeed inverting the nonlinearity and analyze it in detail for a particular case of nonlinear distortion.

In order to maximize the entropy of the observations, we introduced two different parameterization strategies based on polynomial and neural network parameterizations, respectively, and we developed constrained gradient steepest ascend MaxEnt algorithms.

By an extensive experimental set, we explored several possible scenarios, analysing the effect of the type of the sources (uniform or discrete random noise), the length of the sources (*T*), the mixing matrix **A**, the nonlinearities and the number of sources (*N*). We also performed experiments comparing both types of proposed parameterizations, i.e. polynomial and neural network, in order to show pros and cons for each one.

Polynomial parameterization is a very good option when the function to be compensated can be inverted with a polynomial of low order (less than *K* = 12). In this case, the method is extremely fast and converges in a very few number of iterations. Moreover, the order of the inverse function (polynomial) can be adjusted automatically by evaluating the objective function E[log(|**a**^T^**e**|)], as detailed in eq (6). This allows overcoming the problem of tuning parameters, making the method easier to use. On the other hand, neural network parameterization obtains similar results as Gaussianization or Uniformization for continuous uniform random noise, and overcomes those methods for discrete random noise sources. The main advantage of this parameterization is that it can deal with nonlinear functions that are not invertible with a polynomial, obtaining in this case better results than Gaussianization or Uniformization (see Fig 4b). The main drawback of neural network parameterization is that the inversion of nonlinearities is much slower than the polynomial case, about 1000 times slower.

If the number of sources is high, or one source is known to be Gaussian in the case of 2 sources, Gaussianization method works equal or better than MaxEnt because the linear mixture is Gaussian. But in practical situations we do not have access to prior information about the sources, therefore MaxEnt with polynomial parameterization is a good option.

As demonstrated by the experiments, maximizing entropy allow us to decouple estimations of the nonlinear part from the linear one. Therefore, in the PNL source separation scenario, we can apply this strategy to linearize the system and revert the problem to a (linear) blind source separation scenario. As the nonlinearities are inverted independently in each channel, this part can be solved in parallel. Then, any source separation algorithm for linear mixture can be used in order to recover the original observations. This procedure can also be applied for solving Wiener systems that, as it has been explained above, are equivalent to PNL mixtures and therefore can be processed with similar techniques.

Further work can be done, especially concerning the choice of the parametric form for the nonlinear functions. Polynomials are interesting for their simplicity and the linearity with respect to the parameters. For many monotonic functions only a few parameters are required, but the number of parameters can increase dramatically for functions with very large slopes. Then, splines or radial basis functions (RBF) could be a good alternative to consider.

## Appendix: Proofs

### Proof of Theorem 1

*We need to prove that*, *under the condition of* eq (1), *the following equation holds true*:

$$H\left(\bar{E}\right)<H\left(X\right)$$ (17)

*where $\bar{E}$ is the normalized version of variable E, i.e. $\bar{E}=E/\sigma_{E}$*, *with $\sigma_{E}=\sqrt{\text{E}\left[E^{2}\right]}$ is the standard deviation of the output variable E. By using a property of the entropy we obtain*

$$H\left(\bar{E}\right)=H\left(X\right)+\text{E}\left[\text{log}\left(f^{\prime }\left(X\right)\right)\right]-\text{ log}\left(\sigma_{E}\right).$$ (18)

*Thus*, *by proving that E*[*log*(*f*′(*X*))] *< log*(*σ*_*E*_) *will suffice to prove this theorem*.

*The condition of* eq (1) *implies that*

$$\begin{array}{c} \text{E}\left[f^{\prime }\left(X\right)\right]<\sigma_{E}\\ \text{log}\left(\text{E}\left[f^{\prime }\left(X\right)\right]\right)<\text{log}\left(\sigma_{E}\right), \end{array}$$ (19)

*and*, *by applying the Jensen inequality to the last line in* eq (19), *we arrive at*:

$$\text{E}\left[\text{log}\left(f^{\prime }\left(X\right)\right)\right]<\text{log}\left(\sigma_{E}\right)$$ (20)

*which implies that* E[*log*(*f*′(*X*))] *< log*(*σ*_*E*_) *and completes the proof*.

### Proof of Corollary 1

*Here*, *we study the conditions under which* eq (1) *holds true*, *for the particular case of having the nonlinear transform f*(*x*) = *x*^3^ + *βx*. *In this case*, *it is straightforward to evaluate the left and right hands of* eq (1) *as follows*:

$$E^{2}\left[f^{\prime }\left(X\right)\right]={\text{E}}^{2}\left[3X^{2}+\beta \right]={\left(3+\beta \right)}^{2}$$ (21)

*and*

$$\text{E}\left[f^{2}\left(X\right)\right]=\text{E}\left[X^{6}+2\beta X^{4}+\beta^{2}X^{2}\right]=\mu_{6}+2\beta \mu_{4}+\beta^{2}.$$ (22)

*By putting together the above two equations into* eq (1), *we obtain the following general condition*:

$$2\beta (\mu_{4}-3)+(\mu_{6}-9)>0.$$ (23)

*This equation states the conditions on the moments μ*_4_, *μ*_6_ *and the parameter β that make the sufficient condition of* eq (1) *holds true*.
